# Silencing of IQGAP1 by shRNA inhibits the invasion of ovarian carcinoma HO-8910PM cells in vitro

**DOI:** 10.1186/1756-9966-27-77

**Published:** 2008-11-27

**Authors:** Pei-Xin Dong, Nan Jia, Zhu-Jie Xu, Ying-Tao Liu, Da-Jin Li, You-Ji Feng

**Affiliations:** 1 Hospital and Institute of Obstetrics and Gynecology, Fudan University Shanghai Medical, College, Shanghai, PR China; 2 Department of Cardiology, Shanghai Jiao Tong University Affiliated First People's Hospital, Shanghai, PR China; 3 Department of Cardiovascular Medicine Hokkaido University Graduate School of Medicine, Sapporo, Japan

## Abstract

**Background:**

IQGAP1 is a scaffolding protein and overexpressed in many human tumors, including ovarian cancer. However, the contribution of IQGAP1 to invasive properties of ovarian cancer cells remains unknown. Here, we investigated the effect of IQGAP1-specific short hairpin RNA (shRNA) expressing plasmids on metastatic potential of ovarian cancer HO-8910PM cells.

**Methods:**

We used RT-PCR and Western blot analysis to characterize expression of IQGAP1 in three human ovarian cancer-derived cell lines SK-OV-3, HO-8910 and HO-8910PM. We then determined whether expression of endogenous IQGAP1 correlated with invasive and migratory ability by using an in vitro Matrigel assay and cell migration assay. We further knocked down IQGAP1 using shRNA expressing plasmids controlled by U1 promoter in HO-8910PM cells and examined the proliferation activity, invasive and migration potential of IQGAP1 shRNA transfectants using MTT assay, in vitro Matrigel-coated invasion assay and migration assay.

**Results:**

IQGAP1 expression level seemed to be closely associated with the enhanced invasion and migration in ovarian cancer cell lines. Levels of both IQGAP1 mRNA and protein were significantly reduced in HO-8910PM cells transfected with plasmid-based IQGAP1-specific shRNAs. RNAi-mediated knockdown of IQGAP1 expression in HO-8910PM cells resulted in a significant decrease in cell invasion and migration.

**Conclusion:**

Our findings support the hypothesis that IQGAP1 promotes tumor progression and identify IQGAP1 as a potential therapeutic strategy for ovarian cancer and some other tumors with over-expression of the IQGAP1 gene.

## Background

Ovarian carcinomas are high aggressive tumors associated with high mortality and morbidity in gynecology [1]. The poor prognosis of the patients with advanced stage ovarian cancerovarian cancer is largely attributed to the advanced stage of disease at the time of diagnosis. Despite the therapeutic advance, the 5-year survival rate for patients with advanced stage ovarian cancer still remains at 15–30% [2]. These poor outcomes are due mainly to the progression and metastasis of the disease after the standard surgical treatment. Clearly, a better understanding of the molecular mechanisms underlying the progression of ovarian carcinomas is needed to control the disease.

IQGAP1 is a scaffolding protein and binds to a diverse array of signaling and structural molecules, such as F-actin [3], calmodulin [4], CLIP-170 [5], E-cadherin [6] and small GTPases (Cdc42 and Rac1) [7]. Previous studies have shown that IQGAP1 expression is up-regulated in human colorectal carcinoma, especially in invasion front [8]. In addition, IQGAP1 has been suggested to regulate Salmonella invasion through interactions with actin, Rac1, and Cdc42 [9]. We have also reported that IQGAP1 was overexpressed in ovarian adenocarcinomas compared with adenomas and borderline tumors and its expression significantly correlated with poor prognosis in patients with ovarian carcinomas [10]. These lines of evidence have suggested the functional linkage between IQGAP1 and ovarian cancer invasion. However, the exact mechanisms by which IQGAP1 regulates invasion and metastasis of ovarian carcinomas have not yet been elucidated.

RNA interference (RNAi) was a recently discovered antiviral mechanism in plants and invertebrates induced by small double-stranded RNA (dsRNA), which will lead to sequence-specific gene silencing at the post-transcriptional level [11]. Short hairpin RNAs (shRNAs) driven by polymerase III promoters have been investigated as an alternative strategy to suppress gene expression more stably, and such constructs with well-defined initiation and termination sites have been used to produce various small dsRNA species that inhibit the expression of genes with diverse functions in mammalian cell lines [12].

In this study, we examined the effects of IQGAP1 silencing on cell invasion and migration, and explored it as a therapeutic target for metastasis of human ovarian carcinoma cells. We showed that a significant reduction in IQGAP1 expression can markedly inhibit the invasion and migration potentials of ovarian cancer HO-8910PM cells. Thus, our results provide new evidence of the potential use of IQGAP1-targeted RNAi as a novel way to reduce tumor progression of patients with ovarian cancer.

## Methods

### Cell culture

The human ovarian cancer cell line SK-OV-3, HO-8910 (a human ovarian cancer cell line established from a patient with poorly-differentiated serous carcinoma) and HO-8910PM (a highly metastatic cell line derived from HO-8910) [13] were grown in RPMI 1640 medium (Gibco) supplemented with 10% of fetal bovine serum (Cambrex Bio Science, Walkersville, MD). The cells were maintained at 37°C in a humidified atmosphere of 5% CO_2_.

### IQGAP1 silencing

shRNA plasmids (KH0073P) that specifically knock out human IQGAP1 (NM_003870) were obtained from Bioscience Corporation. The oligonucleotide sequence was as follows: 5'-CAACGACATTGCCAGGGATAT-3' (Clone 1), 5'-AAACTGACCCTGTGGATATTT-3' (Clone 2), 5'-ACAGATTCCTGCAGCTAAACT-3' (Clone 3), 5'-GCATGCTGCAGCTAAACT-3' (Clone 4) and 5'-GGAATCTCATTCGATGCATAC-3' (scrambled control). HO-8910PM cells at 80% confluency were transfected with Lipofectamine PLUS Reagent (Invitrogen, Carlsbad, CA) according to the manufacturer's instructions. For establishing stable clones, the transfected cells were selected in RPMI 1640 medium containing puromycin (Sigma, USA) at 1 μg/ml 48 h post-transfection. Selected clones of HO-8910PM cells were expanded into clone 1-, clone 2-, clone 3-, clone 4-HO-8910PM-shIQGAP1 cells and scrambled control-transfectants (HO-8910PM-shRNA negative), respectively.

### MTT assay

For measurements of cell proliferation rates, 1 × 10^3 ^cells/100 μl medium were plated into each well of 96-well plates. After 24, 48, 72 or 96 h incubation, 10 μl of MTT solution (Cell counting kit-8, Dojindo, Kumamoto, Japan) was added into each well, and plates were incubated for 4 h at 37°C, and 450 nm UV absorbance of each sample was measured in a microplate reader. Assay was done in triplicate wells, and each experiment was repeated three times.

### In vitro Matrigel invasion assay

Matrigel invasion assay was performed using a 24-well invasion chamber system (BD Biosciences, Bedford, MA) with Matrigel membrane (8.0-μm pore), as described in our previous report [14]. Briefly, each 750 μl of RPMI 1640 medium supplemented with 20% FBS and 10 μg/ml of bovine fibronectin (chemoattractant) was placed in the lower compartment of the chamber. In the prewarmed and rehydrated upper compartment, 2 × 10^4 ^cells in 500 μl of RPMI 1640 medium supplemented with 20% FBS were added, and the cells were allowed to migrate through the intermediate membrane for 24 h at 37°C. Membranes were then fixed with 10% neutral-buffered formalin and stained in 5% Giemsa solution. The cells attached to the lower side of the membrane were counted in 10 high-powered (×200) fields under a microscope. Assays were done in triplicate for each experiment, and each experiment was repeated three times.

### In vitro cell migration assay

This migration assay was a modification of the assay described previously [15], which measured cell migration through an 8.0-μm pored membrane (BD Biosciences, Bedford, MA). In the lower chamber, 600 μl of RPMI 1640 medium containing 20% FBS and 10 μg/ml of bovine fibronectin was placed. 2 × 10^4 ^cells in 100 μl of RPMI 1640 medium supplemented with 20% FBS were placed in the upper chamber. After 6 h-incubation, the number of migrated cells (lower side of the membrane) was counted as described above. Assays were done in triplicate for each experiment, and each experiment was repeated three times.

### Reverse transcription-polymerase chain reaction (RT-PCR)

The procedures for reverse transcription-polymerase chain reaction (RT-PCR) were essentially as usual. The forward and reverse primers corresponding to human IQGAP1 were 5'-ACCGTGGACCCAAAGAAC-3' (forward), 5'-CTTCCCGTAGAACTTTTTGTTG-3' (reverse) [16]. Beta-actin mRNA was amplified with forward (5'-TTGCCGACAGGATGCAGAA-3') and reverse (5'-GCCGATCCACACGGAGTACT-3') primers in a similar fashion. All RNA was extracted by using Trizol reagent (Invitrogen) according to the manufacture's instructions. Total RNA was reverse transcribed in 20 μl reaction system using Superscript First-Strand Synthesis Kit for RT-PCR (Invitrogen) under conditions described by the supplier. The PCR cycling program was 94°C for 5 minutes, then 30 cycles of 94°C for 30 seconds, 55°C for 60 seconds, and 72°C for 30 seconds, and a final extension at 72°C for 10 min. The RT-PCR products obtained were electrophoresed through a 2% agarose gel with ethidium bromide.

### Western blot analysis

Whole cellular protein was obtained with M-Per Mammalian Protein Extraction Reagent (Pierce, Rockford, IL). The aliquots were separated on SDS-PAGE (10%) and transferred to nitrocellulose membranes. Antigen-antibody complexes were detected ECL blotting analysis system (Amersham Biosicences Inc.). The IQGAP1 antibody (H00008826-M01) was purchased from Novus Biologicals. Anti-b-actin antibody was obtained from Sigma Biotechnology. The secondary antibody was sc-2005, a goat anti-mouse immunoglobulin G, horseradish peroxidase (HRP) conjugate.

### Statistical analysis

Statistical analyses were performed using SPSS statistical software (SPSS Inc., Chicago, Illinois). Student's t-test was adopted. Significance was defined as *P *< 0.05.

## Results

### IQGAP1 expression in ovarian cancer cell lines

**Figure 1:**
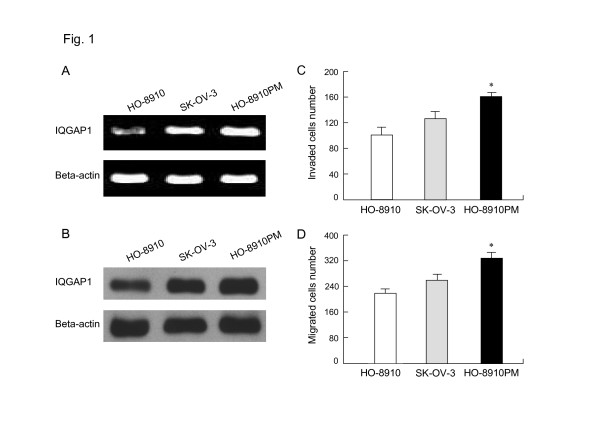
**shows that IQGAP1 expression and cell invasive phenotype in ovarian cancer cell lines**. (a) RT-PCR analysis for IQGAP1 using total RNA form HO-8910, SK-OV-3 and HO-8910PM cells.   (b) Western blot analysis for IQGAP1 in whole-cell lysates from the indicated cell lines.   (c) High IQGAP1 expression associated with enhanced invasive potential of ovarian cancer cells. HO-8910, SK-OV-3 and HO-8910PM cells were seeded onto a Matrigel-coated invasion chamber and the number of invading cells was determined as described before.  *, P < 0.05 versus SK-OV-3 or HO-8910 cells.   (d) High IQGAP1 expression correlated with enhanced migratory potential of ovarian cancer cells. HO-8910, SK-OV-3 and HO-8910PM cells were seeded onto a Boyden chamber and the number of migrating cells was determined as described before.  *, P < 0.05 versus SK-OV-3 or HO-8910 cells.

We used RT-PCR and Western blot analysis to characterize expression of IQGAP1 in three human ovarian cancer-derived cell lines (SK-OV-3, HO-8910 and HO-8910PM). IQGAP1 mRNA and protein was detectable in all these three cell lines, with highest expression in HO-8910PM, moderate expression in SK-OV-3 and weak expression in HO-8910 cells (Fig. 1a and 1b).

### IQGAP1 expression and invasive phenotype in ovarian cancer cell lines

IQGAP1 is thought to mediate tumor cell invasion and migration in several types of cancer. To assess the role of IQGAP1 in conferring invasive properties to cells derived from ovarian cancer, we first determined whether expression of endogenous IQGAP1 correlated with invasive and migratory ability by using an in vitro Matrigel assay and cell migration assay. In contrast to SK-OV-3 and HO-8910 cells with markedly reduced endogenous IQGAP1 expression, HO-8910PM cells expressed high levels of IQGAP1 protein displayed much higher metastatic potential after 24-h incubation. The number of HO-8910PM cells that passed through the Matrigel-coated membrane was 1.3 times and 1.6 times larger than the number of SKOV-3 and HO-8910 cells, respectively (*P *< 0.05) (Fig. 1c). After 6 h-incubation, HO-8910PM cells showed much stronger migratory activities compared with SK-OV-3 and HO-8910 cells (*P *< 0.05) (Fig. 1d). Thus, these findings suggest that IQGAP1 expression level seemed to be closely associated with the enhanced invasion and migration in ovarian cancer cell lines.

### Gene silencing for IQGAP1 gene

**Figure 2:**
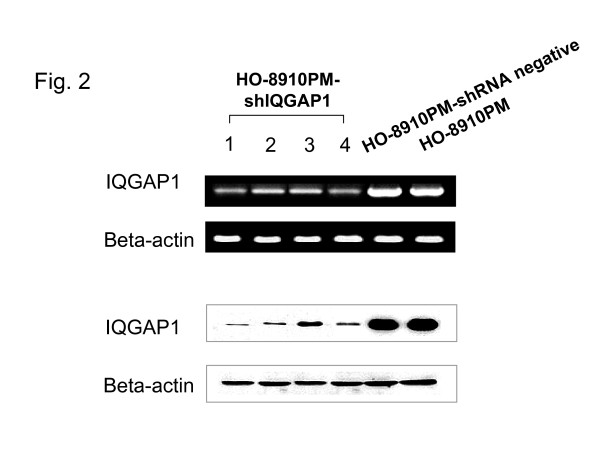
indicates that IQGAP1 mRNA and protein levels in HO-8910PM-shIQGAP1, HO-8910PM-shRNA negative and un-transfected HO-8910PM cells were determined by RT-PCR and Western blot analysis, respectively.

To further investigate whether IQGAP-1 can induce cancer cell invasion and migration, we knocked down IQGAP1 using shRNA expressing plasmids controlled by U1 promoter (Clone 1, 2, 3 and 4) in HO-8910PM cells and selected by growth in the presence of puromycin. We first examined the knock-down efficiencies of different IQGAP1 shRNAs using Western blot analysis. HO-8910PM-shIQGAP1 cells (Clone 1, 2, 3 and 4) showed a significant decrease in IQGAP1 mRNA and protein expression when compared with HO-8910PM-shRNA negative cells or un-transfected HO-8910PM cells (Fig. 2). The above results demonstrated that the expression of IQGAP1 could be down-regulated specifically and effectively by specific IQGAP1 shRNA.

### Effects of IQGAP1 specific shRNA on cell proliferation activity

**Figure 3:**
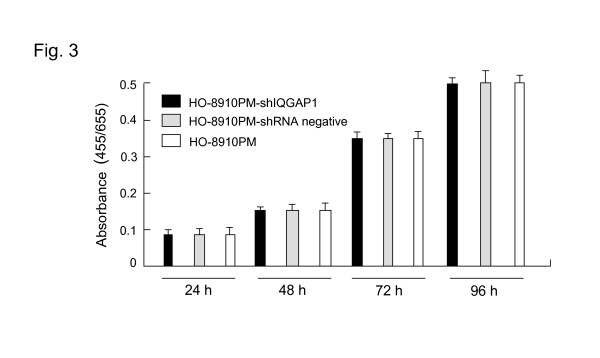
**shows that proliferation of HO-8910PM cells stably expression shRNA**. Proliferation of HO-8910PM-shIQGAP1 (clone 1), HO-8910PM-shRNA negative and un-transfected HO-8910PM cells were analyzed by MTT assay as described earlier.

The proliferation activity of tumor cell is important in invasion and metastasis of tumor. We examined cell proliferation activity of HO-8910PM-shIQGAP1 (clone 1), HO-8910PM-shRNA negative cells and un-transfected HO-8910PM cells using MTT assay. The proliferation rates of HO-8910PM-shIQGAP1 cells were similar to HO-8910PM-shRNA negative and un-transfected HO-8910PM cells (Fig. 3). The results showed that knockdown of IQGAP1 in the HO-8910PM cells by shRNA did not change the cell proliferation activity in vitro.

### Silencing of IQGAP1 inhibited ovarian cancer cell invasion in vitro

**Figure 4:**
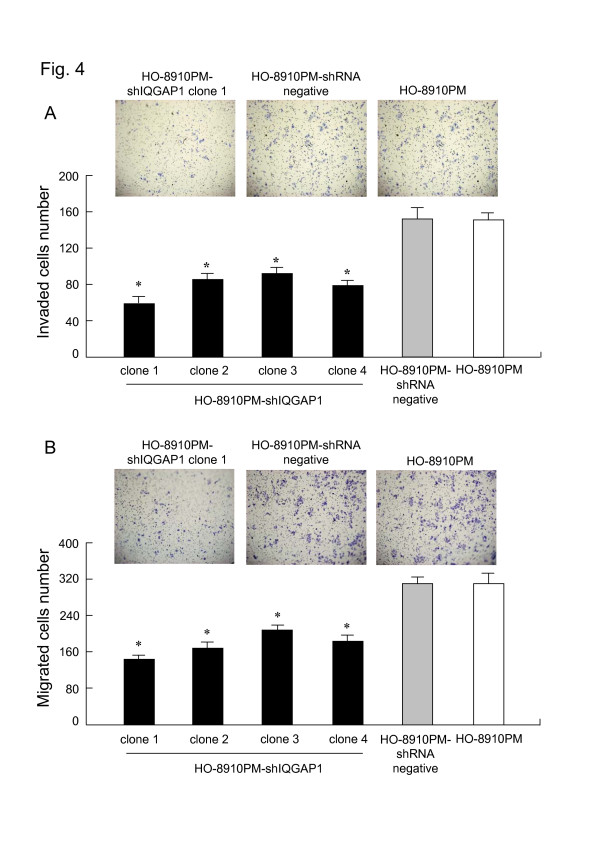
**indicates that silencing of IQGAP1 inhibited ovarian cancer cell invasion and migration in vitro**. (a) Suppression of IQGAP1 hampered transmembrane migration ability of HO-8910PM cells. HO-8910PM-shIQGAP1, HO-8910PM-shRNA negative and un-transfected HO-8910PM cells were seeded onto a Matrigel-coated invasion chamber and the number of invading cells was determined as described before.  *, P < 0.05 versus HO-8910PM-shRNA negative or un-transfected HO-8910PM cells.     (b) Suppression of IQGAP1 decreased migration of HO-8910PM cells. HO-8910PM-shIQGAP1, HO-8910PM-shRNA negative and un-transfected HO-8910PM cells were seeded onto a Boyden chamber and the number of migrating cells was determined as described before.  *, P < 0.05 versus HO-8910PM-shRNA negative or un-transfected HO-8910PM cells.

Invasion of basement membranes by tumor cell is thought to be a critical event in the cascade of metastasis. It has been shown that over-expression of IQGAP1 enhances the capacity of human breast adenocarcinoma MCF-7 cell for transmembrane migration by up-regulating the expression active CDC42 and Rac1 [17]. To evaluate whether silencing of IQGAP1 contributes to the reduced invasive behavior of ovarian cancer cells, we examined the invasive potential of IQGAP1 shRNA transfectants using an in vitro Matrigel-coated invasion assay. HO-8910PM-shIQGAP1 cells displayed a significantly lower transmembrane migration activity as compared to HO-8910PM-shRNA negative and un-transfected HO-8910PM cells (Fig 4a). This result suggested that suppression of IQGAP1 by shRNA resulted in a significant decrease in the invasiveness of ovarian cancer cells.

### Suppression of IQGAP1 decreased cell mobility

Active cell motility is another rate-limiting step of tumor cell invasion. To explore whether shRNA-induced gene silencing of IQGAP1 affect the motility properties of HO-8910PM cells, we also compared the cell motility using an in vitro cell migration assay. After 6 h-incubation, HO-8910PM-shIQGAP1 cells showed a significantly lower cell invasion activity as compared to HO-8910PM-shRNA negative and un-transfected HO-8910PM cells (Fig. 4b), suggesting that suppression of IQGAP1 decreases cell motility, therefore, supporting a important role for IQGAP1 in the metastasis of ovarian cancer cells [10].

## Discussion

IQGAP1 is a scaffolding protein that binds to a diverse array of signaling and structural molecules. Accumulated evidences have demonstrated that IQGAP1 may be critical for conferring invasive potential in various human cancers [8,17]. We have reported that of IQGAP1 was a novel marker of poor outcome in patients with ovarian carcinomas [10]. In the present study, we have shown that ovarian cancer cell line HO-8910PM, which expresses high levels of endogenous IQGAP1, showed extremely high invasive abilities through the reconstituted basement membrane Matrigel. Furthermore, gene silencing for IQGAP1 in HO-8910PM cells resulted in reduced cell migration. This was consistent with previous findings that down-regulation of IQGAP1 by IQGAP1-specific siRNAs effectively blocks HA-CD44-stimulated SK-OV-3 ovarian tumor cell migration [18]. More importantly, we first demonstrated that knockdown of IQGAP1 also led to attenuated Matrigel invasiveness, suggesting that therapies targeting IQGAP1 activity may prove efficacious for treating ovarian cancer metastasis. Moreover, accumulating evidences have shownthat non-viral vectors will be an attractive alternative to viral vectors due to their safety, versatility and ease of preparation and scale-up [19]. Intravenous delivery of liposome-mediated nonviral DNA has also been proved to be less toxic than intraperitoneal delivery in mice [20]. Therefore, we will further investigate the effects of IQGAP1-shRNA plasmids by intravenous administration on ovarian cancer metastasis in animal models.

In addition, recent study demonstrated that IQGAP1 stimulated proliferation of human breast epithelial cells [21]. However, our result indicated that IQGAP1-specific shRNA did not influence cell proliferation in HO-8910PM cells, suggesting that IQGAP1 may induce cell proliferation rate in a cell type-dependent mechanism. Collectively, these findings suggest that IQGAP1 plays an important role in the invasion and metastasis of ovarian cancer cells.

The acquisition of invasive potential by tumor cells is undoubtedly a complex process. Degradation of extracellular matrix and basement membrane, promotion of cell motility and enhanced tumor cell proliferation are thought to be essential steps. It has been shown that IQGAP1 captures and stabilizes microtubules through the microtubule-binding protein CLIP-170 near the cell cortex, leading to establishment of polarized cell morphology and directional cell migration [5]. Moreover, IQGAP1 regulates Salmonella invasion through interactions with actin, Rac1, and Cdc42 [9]. To gain full understanding of molecular mechanisms by which IQGAP1 regulates cell invasion, DNA microarray analysis was further needed to identify key genes involved in IQGAP1-dependent ovarian cancer cell invasiveness.

Inhibiting the expression of pathogenic genes by shRNA-induced RNA interference has proved to be a successful approach to reducing the malignance of different tumor cells [22]. In the present study, we selectively silenced IQGAP1 expression in human ovarian cancer cells by exploiting RNAi technology. Furthermore, selective inhibition of IQGAP1 significantly reduced metastatic potential of IQGAP1-expressing human ovarian cancer cell in vitro by affecting multiple aspects of tumor invasion, raising the possibility that RNA interference against IQGAP1 is an effective strategy for anti-metastasis of IQGAP1 expressing ovarian cancer cells.

Ovarian cancer is the fifth most common cancer, and it is the leading cause of death from all types of gynecologic cancer. It is urgent to develop new therapeutic strategies for ovarian cancers. In this report, the knockdown of IQGAP1 expression by RNAi can successfully reverse the malignant behaviors of ovarian cancer cells, implicating that IQGAP1 may be a new candidate of drug target for treatment of ovarian cancers.

## Competing interests

The authors declare that they have no competing interests.

## Authors' contributions

PXD designed research; PXD, NJ, ZJX and YTL carried out the molecular genetic studies; PXD, DJL and YJF analyzed data; PXD wrote the paper. All authors read and approved the final manuscript.
